# *α*-Amylase inhibitor-1 gene from *Phaseolus vulgaris *expressed in *Coffea arabica *plants inhibits α-amylases from the coffee berry borer pest

**DOI:** 10.1186/1472-6750-10-44

**Published:** 2010-06-17

**Authors:** Aulus EAD Barbosa, Érika VS Albuquerque, Maria CM Silva, Djair SL Souza, Osmundo B Oliveira-Neto, Arnubio Valencia, Thales L Rocha, Maria F Grossi-de-Sa

**Affiliations:** 1 EMBRAPA Recursos Genéticos e Biotecnologia, Brasília-DF, Brazil; 2 Pós-graduação em Ciências Genômicas e Biotecnologia, UCB, Brasília-DF, Brazil; 3 Facultad de Ciencias Agropecuarias, Universidad de Caldas, Manizales, Colombia; 4 Dept de Biologia Celular, Universidade de Brasília, Brasília-DF, Brazil

## Abstract

**Background:**

Coffee is an important crop and is crucial to the economy of many developing countries, generating around US$70 billion per year. There are 115 species in the *Coffea *genus, but only two, *C. arabica *and *C. canephora*, are commercially cultivated. Coffee plants are attacked by many pathogens and insect-pests, which affect not only the production of coffee but also its grain quality, reducing the commercial value of the product. The main insect-pest, the coffee berry borer (*Hypotheneumus hampei*), is responsible for worldwide annual losses of around US$500 million. The coffee berry borer exclusively damages the coffee berries, and it is mainly controlled by organochlorine insecticides that are both toxic and carcinogenic. Unfortunately, natural resistance in the genus *Coffea *to *H. hampei *has not been documented. To overcome these problems, biotechnological strategies can be used to introduce an α-amylase inhibitor gene (*α-AI1*), which confers resistance against the coffee berry borer insect-pest, into *C. arabica *plants.

**Results:**

We transformed *C. arabica *with the α-amylase inhibitor-1 gene (α*-AI1*) from the common bean, *Phaseolus vulgaris*, under control of the seed-specific phytohemagglutinin promoter (PHA-L). The presence of the α*-AI1 *gene in six regenerated transgenic T1 coffee plants was identified by PCR and Southern blotting. Immunoblotting and ELISA experiments using antibodies against α-AI1 inhibitor showed a maximum α-AI1 concentration of 0.29% in crude seed extracts. Inhibitory *in vitro *assays of the α-AI1 protein against *H. hampei *α-amylases in transgenic seed extracts showed up to 88% inhibition of enzyme activity.

**Conclusions:**

This is the first report showing the production of transgenic coffee plants with the biotechnological potential to control the coffee berry borer, the most important insect-pest of crop coffee.

## Background

Coffee is one of the most valuable primary products in world trade, and its cultivation, processing, transportation and marketing provide employment for around 25 million people worldwide [1]. Furthermore, this culture is crucial to the economies of many developing countries, and its international trade reaches up to US$70 billion per year [2]. World coffee production is around 7 million metric tons, and Brazil is the leading producer [3].

*Coffea arabica *is the main cultivated coffee species (70%) worldwide [4], and its production is commonly affected by different insect-pests. Among these pests, the most damaging is the coffee berry borer (CBB), *Hypothenemus hampei *(Ferrari, 1867) (Coleoptera: Curculionidae). CBB attacks and feeds on coffee berries, decreasing the quality and the flavour of the grain coffee and causing world-wide monetary losses of around US$500 million annually [5]. A major part of the insect life cycle is spent inside the coffee beans, making control very difficult [6]. Current methods used to control CBB are based on cultural, biological and predominantly chemical approaches. The use of the insecticide ENDOSULFAN is very widespread; however, CBB with high levels of resistance to ENDOSULFAN have already been detected and reported in New Caledonia [7]. In addition, of the 115 species in genus Coffea described, none have shown any natural resistance against CBB [8], making it unlikely that control of this insect can be achieved by plant breeding alone.

Biotechnology may be a promising alternative for *H. hampei *control. Several genes are potentially available for this purpose, including Bt toxins, digestive enzyme inhibitors, chitinases and lectins [9-15]. With respect to the enzyme inhibitor class, the expression of alpha-amylase inhibitors (*α*-AI) from both scarlet runner bean (*Phaseolus coccineus*) and common bean (*Phaseolus vulgaris*) has been shown to be effective in transgenic plants, showing high protection against seed weevils in pea [16,17], azuki bean [18], chickpea [19,20], and cowpea [21]. With pea, complete protection against the pea weevil *Bruchus pisorum *was shown under field conditions [22]. In all of these experiments, expression of the α-AI coding region was driven by the seed-specific promoter of *P. vulgaris *phytohemagglutinin. Interestingly, both amylase inhibitors, *α-AI1* from *P. vulgaris* and α-AI1-like amylase inhibitor from wild accessions of scarlet runner bean have shown to inhibit the α-amylases from *H. hampei *[23,24].

Here we describe the introduction of an expression cassette carrying the *α-AI1* gene under phytohemagglutinin seed-specific promoter control in *C. arabica* plants using biolistics, followed by regeneration of coffee plants.  According to the molecular characterisation and *in vitro *assays, extracts from the transgenic plants with a relatively high level of expressed *α*-AI1 protein are active against coffee berry borer α-amylases, indicating that this transformation event with *α-AI1 *represents a promising method that can be applied to the control of CBB.

## Results and discussion

### Transformation of *C. arabica *plants

**Figure 1:**
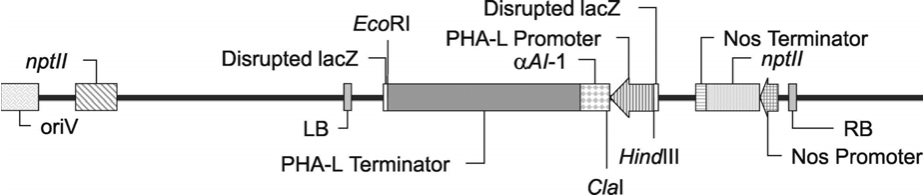
**Linear representation of the plasmid vector pBIN19*α*-AI1**. The *α-AI1 *is under control of the phytohemagglutinin (PHA-L) promoter and terminator (PHA-L Terminator); *Nos*, Nopaline synthase.

**Figure 2:**
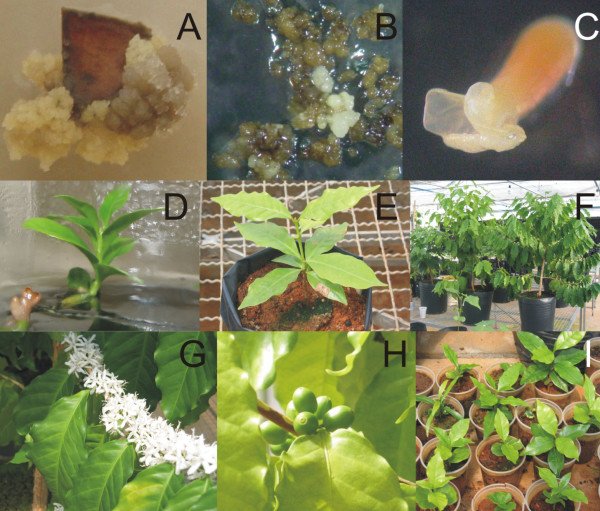
***Coffea arabica *transformation steps**. A) Friable callus used in the transformation. (B) Callus after bombardment under kanamycin selection. (C) Regenerating embryo. (D) Small coffee plant under kanamycin selection. (E) Plant transferred to the greenhouse. (F) Coffee plants positive for the *α-AI1 *gene. (G) Flowers of the transformed coffee plant. (H) Transgenic fruits, 10 weeks after flowering. (I) T1 generation from plant 3.

*C. arabica *plants were transformed using particle bombardment with vector pBIN19*α*-AI1. After 8 months of in vitro culture and selection of bombarded *C. arabica *calli had been bombarded (Figures 1, 2A, B and 2C), 26 plantlets were obtained. All displayed normal subsequent development (Figures 2D and 2E). Six positive plants for *α-AI1 *gene were maintained in the greenhouse (Figure 2F) and after two years the first flowers appeared, followed by fruit development (Figures 2G and 2H), like occurs in non transformed coffee plants. Out of the six plants, three were not able to produce fruits after three years of greenhouse cultivation. Sterility in transgenic plants may be caused by somaclonal variation, generated as a result of tissue culture or caused by the position of the transgene in the genome. Similar observations were found in other transgenic plants such as soybean and rice [25].

Transgenic seeds of T0 plants numbered 1, 2 and 3 were collected and used for molecular characterisation, an *in vitro *inhibitory assay, and the production of T1 plants from plants 2 and 3. The coffee beans showed a normal level of germination, ranging from 40% (T1 from plant 3 of T0 generation) to 70% (T1 from plant 2 of T0 generation), in agreement with the data shown by Valio [26], which indicates that the expression of α-AI1 did not interfere with germination and seedling growth (Figure 2I). The genomic DNA from all eleven germinated T1 plants was evaluated using PCR.

### Molecular characterisation of the transformed plants

**Figure 3:**
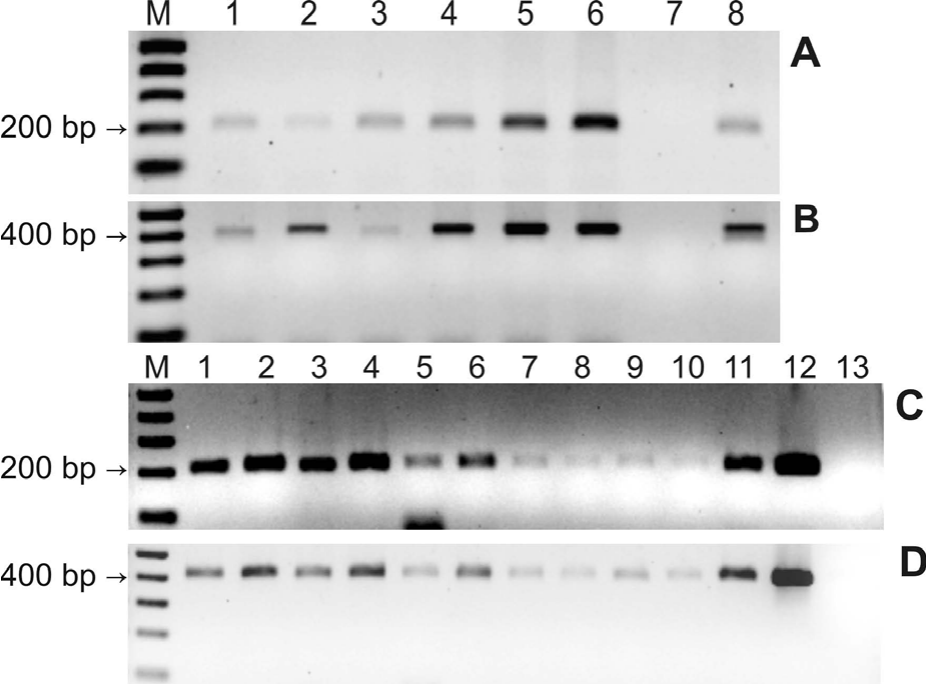
**PCRs of positive *C. arabica *plants, showing *npt*II and *α-AI1 *amplifications **PCRs from T0 (A and B) and T1 plants (C and D). **A**, 204-bp band from the *α-AI1 *gene of T0 plants. **B**, 411-bp band from the *npt*II gene of T0 plants. M, 1 kb plus leader; 1 - 6, T0 transformed coffee plants; 7, non-transgenic coffee plant; 8 positive control (pBIN19*α*-AI1 vector). **C**, 204-bp band from the *α-AI1 *gene of T1 plants. **D**, 411-bp band from the *npt*II gene of T1 plants. M, 1 kb plus leader; 1 - 11, positive T1 plants; 12, positive control (pBIN19*α*-AI1 vector); 13 non-transgenic coffee plant.

Six PCR positive coffee plants (T0) were obtained after biolistic transformation, showing the presence of both *npt*II and *α-AI1 *genes (Figures 3A and 3B). The transformation efficiency obtained in this work was 23.1% (6 positive: 20 negative plants). Although generation of spontaneous kanamycin resistance is very well documented [27], the low efficiency may also be a consequence of unstable genome insertion.

**Figure 4:**
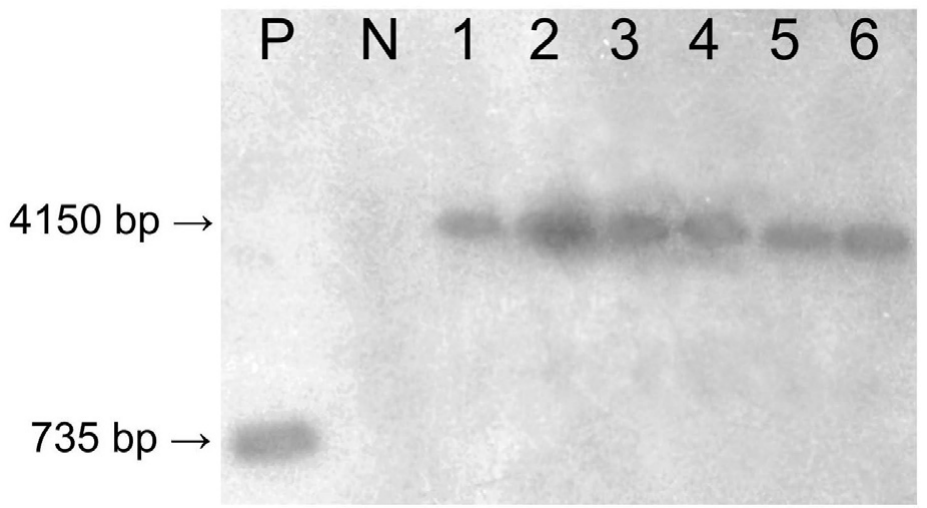
**Southern blot indicating the presence of *α-AI1 *in the genome of *Coffea arabica *T0 plants**. P, positive control (*α-AI1 *Fragment, 735 bp); N, non-transformed coffee plant; 1 - 6, *α-AI1 *positive coffee plants (4150-bp fragment). Plant DNA extracts were digested with the enzymes *Pvu*II, *Eco*RI and *Cla*I at the same time.

The stable integration of the *α-AI1 *gene into the coffee genome was confirmed by Southern blot analysis. The *α-AI1 *probe successfully hybridised with genomic DNA samples from the six positive T0 plants, confirming the PCR results (Figure 4).

As for the T1 plants obtained from seeds of the primary transgenic coffee plants (T0), eleven individuals were obtained and evaluated by PCR. All of them (100%) were positive for the *npt*II and *α-AI1 *genes in these experiments (Figures 3C and 3D), indicating that the genes were transferred by inheritance in transgenic *C. arabica*. Normally, a classic proportion of three transgenic plants for one non-transgenic plant (3:1) is expected for *C. arabica *because it is an allotetraploid crop presenting a regular bivalent pairing of homologous chromosomes [28]. In our results we did not observe a 3:1 proportion, possibly due to the low number of T1 individuals analysed. Alternatively, this proportion of positive T1 plants could be generated by a T0 plant with more than one insertion.

### Analysis of expressed *α*-AI1 in transgenic coffee seeds

**Figure 5:**
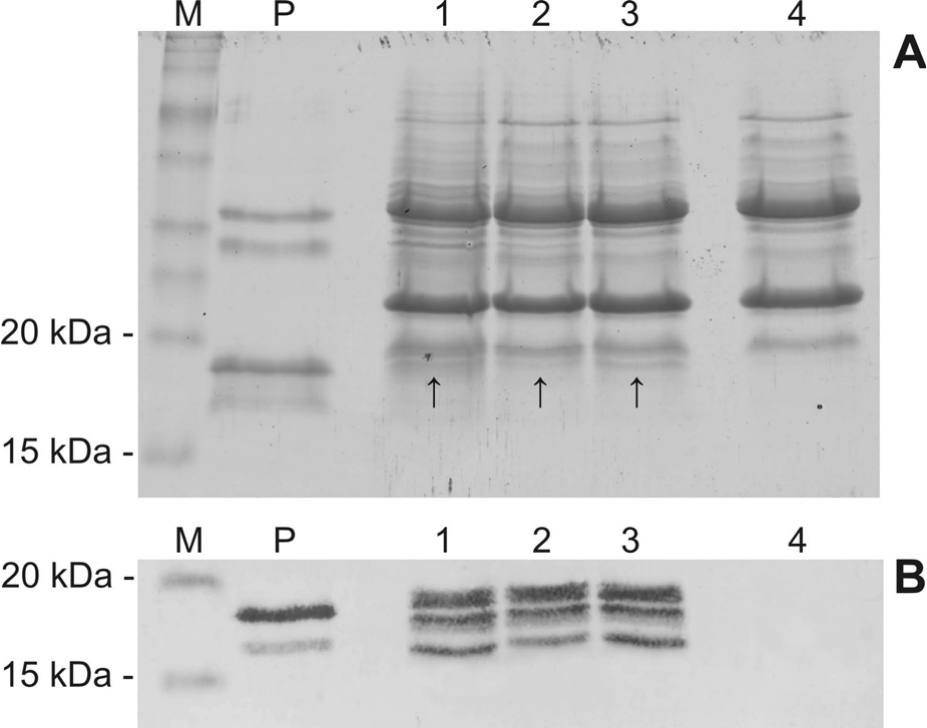
***α*-AI1 expression in transgenic coffee beans from T0 plants**. **A**. SDS-Polyacrylamide gel (15%) stained with Coomassie Brilliant Blue. M, Benchmark™ prestained protein ladder (Invitrogen); P, purified *α*-AI1 from *Phaseolus vulgaris *seeds; 1-3, total seed protein extract from transgenic coffee plants. The arrows indicate possible expressed *α*-AI1; 4, total seed protein extract of non-transgenic coffee plant. **B**. Western blot. M, Benchmark™ prestained protein ladder (Invitrogen); P, purified *α*-AI1 from *Phaseolus vulgaris *seeds; 1-3, total seed protein extract from transgenic coffee plants; 4, total seed protein extract of the non-transgenic coffee plant.

To examine α-AI1 protein expression in coffee beans of T0 plants, crude extracts from transformed seeds were analysed using SDS-PAGE and immunoblots (Figures 5A and 3B, respectively). The level of expression was evaluated by ELISA assay (Table 1). The immunoblot showed three positive bands of 19, 17 and 16 kDa that were not present in the extracts of untransformed plants and corresponded to the protein found in *P. vulgaris *seed extracts (Figure 5B lanes 1, 2, 3 and 4). In the common bean, amylase inhibitor-1 is synthesised as a preproprotein, and in its mature form, it consists of two polypeptides of 19 and 14 kDa [29]. Differential processing of the polypeptide and glycan portions of the protein results in a series of bands on immunoblots, especially in transgenic seeds of different species where processing events may be different. For example, as many as 11 different immunoreactive polypeptides were found in tobacco seeds [30]. In transgenic peas expressing *α*-AI1, three polypeptides were visualised on immunoblots [17]. The altered molecular mass of *α*-AI1 in the different transgenic plants may have occured due to a variation in the extent of glycosylation or the processing of the glycans [31]. Even in *P. vulgaris *seeds, the subunits α and β of *α*-AI1 are not always glycosylated in the same way, generating polypeptide heterogeneity [32]. Unexpectedly, differences in glycosylation between the inhibitor isolated from the common bean and from transgenic peas expressing *α*-AI1 resulted in differences in immunogenic properties between the two proteins. The protein made in peas caused immunological responses and inflammation in mice [33]. Nevertheless, no difference in immunogenicity was observed in mice fed with transgenic chickpeas expressing *α*-AI1 [33]. As a consequence, it is difficult to predict if similar immunogenic alterations will occur in an inhibitor made by transgenic coffee plants.

**Table 1:** Concentration of *α*-AI1 in seed protein extracts from three different transgenic coffee plants and *H. hampei *α-amylase inhibition level using *α*-AI1 expressed in these coffee plants.

	*α*-AI1 concentration (%)	Inhibition of *H. hampei α*-amylase
Plant 1	0.14 ± 0.03%	54.38 ± 20.13%
Plant 2	0.29 ± 0.02%	88.86 ± 14.34%
Plant 3	0.03 ± 0.01%	20.61 ± 13.22%
Negative plant	0.02 ± 0.01%	0.94 ± 12.32%

One unit of *H. hampei α*-amylase activity was utilised in the inhibition assay.

Using ELISA assays, we detected different levels of recombinantly expressed *α*-AI1 in the different transformed plants (Table 1). The highest level was found in plant 2 (Table 1), in which *α*-AI1 comprised 0.29% of the total seed protein extract. These variable protein concentration levels in transgenic plants may be explained by several factors, such as somaclonal variation [34,35], mutations in the inserted gene [36], genetic alterations caused by tissue culture [37], different copy numbers of the inserted transgene [25], or by the position of the transgene inside the plant genome [25]. In transgenic chickpeas and peas seeds, higher *α*-AI1 expression levels were observed: 1.0-3.5% in peas [17] and 4.2% in chickpea [19] or 0.72% in chickpea [17,20].

The phytohemagglutinin (PHA-L) promoter used in this work was cloned from the *P. vulgaris *genome and first analysed in tobacco. The promoter showed similar seed-specific expression, in both,  tobacco and beans  [38]. It is important to emphasise the α-AI1 coffee seed-specific expression driven by this promoter, as it decreases the chance of affecting non-target insects. Because pollinators can increase coffee yields by 20.8% and decrease the frequency of pea berries by 27%, the protection of non-target insects is fundamental for coffee crop production [39].

### Inhibition of *H. hampei α*-amylases by *α*-AI1 expressed in transgenic coffee seeds

**Figure 6:**
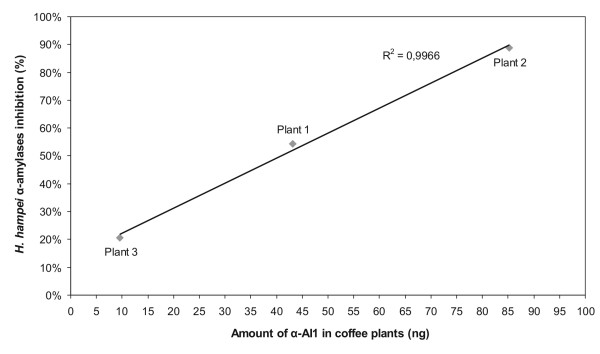
**Comparison between the amount of *α*-AI1 in coffee transgenic plants and the inhibition rate of *H. hampei *α-amylases measured by DNS assay**. In the DNS assay, 30 μg of the total seed protein extract was used, and the *α*-AI1 quantity in these extracts was measured using ELISA.

*In vivo *insect assays described in other works exhibited mortality ranging from 89.8% to 100% [17,20], depending on the inhibitor concentration in each tested plant. The high levels of amylase inhibitor may be related to the fact that in some studies [17], seeds from homozygous plants were used. However, the coffee plants used here were hemizygous. Valencia *et al*. (2000) showed that an inhibitor from common bean inhibits the *H. hampei *gut amylases up to 95%. We tested the effect of extracts from transformed seeds, and the level of inhibition of amylase activity correlates with the level of inhibitor in the common bean (*P. vulgaris*) seeds (Table 1). The plant with the highest amount of α-AI1 inhibitor (Plant 2) exhibited the strongest *H. hampei α-amylases* inhibition, and the inhibition levels reached by each transgenic plant were directly related to the *α*-AI1 concentration (Figure 6).

## Conclusions

The data presented here lead to several important conclusions. From PCR and Southern blot analysis, it was possible to conclude that the *α-AI1 *gene, fused under control of the phytohemagglutinin promoter and terminator, was inserted into the *C. arabica *genome. Both the α-AI1 expression and inhibitor activity were confirmed in coffee seeds. Additional tests will be necessary not only to confirm the *in vivo *efficiency of these transgenic plants against *H. hampei*, but also to analyse the inheritance of the inserted genes through different generations until attainment of a fully homozygous progeny (T3). Moreover, the presence of *npt*II will be evaluated to identify if this gene was inserted in any other locus in the genome, allowing its separation from *α-AI1 *through conventional breeding. Finally, considering the long life cycle of the coffee plant, we consider these transformation events a crucial step that might control *H. hampei*, the main insect pest in coffee.

## Methods

### Plasmid vector construction

Plasmid vector pBIN19αAI-1 (16.6 kb) was constructed using the fragment of a pTA3 plasmid containing the α-amylase inhibitor-1 (*α-AI1*) gene flanked by a phytohemagglutinin (PHA-L) promoter and terminator [29]. The *α-AI1 *expression cassette of pTA3 was digested with *Hind*III and subcloned in the pBIN19 vector [40] using the same restriction site (Figure 1). The PHA-L promoter is seed-specific [38], driving the *α-AI1 *gene expression into the type of tissue attacked by *H. hampei*.

### *Coffea arabica *genetic transformation through bombardment

*Coffea arabica *cv Catuaí Vermelho plants were transformed by bombardment of embryogenic callus, according to the procedures described by Albuquerque *et al*. (2009) [41] and the following details. Explants were obtained from coffee leaf fragments cultivated in C medium [42] modified with 20 μM 2,4-D (C20 medium). After one month of incubation in dark conditions, the produced calli were transferred to fresh medium and cultivated for five additional months. Seven days before the bombardment, embryogenic calli were dispersed over a 0.45 μm Membrane filter in Petri dishes containing C medium with 10 μM 2,4-D (C10 medium). The membranes carrying calli were transferred to C10 medium that contained mannitol (0.5 M) and phytagel (8 g/L) 24 hours before bombardment. After this osmotic treatment, calli were bombarded with tungsten microparticles coated with vector pBIN19*α*-AI1 [29]. Two weeks after transformation, calli were transferred to C10 medium containing the selective agent kanamycin (200 mg/L), and subsequently subcultured in C10 medium containing kanamycin at 300 mg/L and 400 mg/L at one week intervals. Selected calli and somatic embryos were then subcultivated until embryos reached the torpedo stage. Fully developed embryos were cultivated in WPM medium until they become plantlets. Rooted individuals were acclimated and grown in a greenhouse (temperature 27°C ± 3, humidity 75% ± 10) for two years, until the first fruits appeared. The first seeds were used to produce the T1 generation. Two T0 lines (Plants 2 and 3) were selected and ten seeds of each one were planted and maintained in the greenhouse until germination.

### Identification of positive plants through PCR

DNA from the T0 and T1 coffee lines were extracted using the CTAB method modified with the addition of 2% PVP and 2% sodium metabisulfite [43]. The extractions were quantified in a NanoDrop™ spectrophotometer ND-1000 (Thermo Scientific). Before the PCR experiments, 2 μg of DNA from transgenic plants were linearised with the *Eco*RI restriction enzyme to facilitate the primers' alignment. The presence of the kanamycin resistance (*npt*II - 411 bp) and *α-AI1 *genes (204 bp) were detected using the respective primers: *npt*II forward (5'-GAGGCTATTCGGCTATGACTG-3'), *npt*II reverse (5'-TCGACAAGACCGGCTTCCATC-3'), *α-AI1 *forward (5'-GCCTTGGGATGTACACGACT-3') and *α-AI1 *reverse (5-CTCCATTGATAAGCCCCTGA-3'). Both amplification reactions were carried out with 0.6 μg of digested DNA and an initial denaturation at 94°C for 5 min, followed by 30 cycles of denaturation at 94°C for 1 min, annealing at 60°C for 1 min and extension at 72°C for 30 seconds, and a final extension for 10 min at 72°C. DNA from a non-transgenic *C. arabica *plant was used as a negative control, while the pBIN19*α*-AI1 vector served as the positive control. PCR fragments were analysed by electrophoresis on a 1.0% agarose gel stained with ethidium bromide [44]. Eleven plants from T1 generation were evaluated using the same methodology.

### Evaluation of integrated DNA through Southern blot

The Southern blot experiment was carried out with 20 μg of DNA from PCR positive plants digested with the following three restriction enzymes at the same time: *Pvu*II, *Eco*RI and *Cla*I. The enzyme *Cla*I cut only at the beginning of the *α-AI1 *gene (nucleotide 86) and the enzyme *Eco*RI cut only at the end of the phytohemagglutinin terminator, releasing a DNA fragment with 4150 bp The *Pvu*II enzyme has no corresponding restriction site inside of the vector sequence. An *α-AI1 *fragment excised from the pBIN19*α*-AI1 vector, using *Hind*III enzyme, was used as a positive control, and DNA of a non-transformed coffee plant was used as a negative control. The digested samples were submitted to electrophoresis on a 0.8% agarose gel and transferred by capillarity to a Hybond-N membrane (Amersham Biosciences). The probe was constructed using an *α-AI1 *fragment radioactively labelled with the Ready-To-Go™ DNA Labeling Beads kit and [α^32^P] dCTP, both from GE Healthcare. Pre-hybridisation and hybridisation were performed as described by [44].

### Detection of expressed α-AI-1 in seeds of *Coffea arabica*

The α-AI1 expression levels in the coffee beans was analysed by SDS-PAGE and western blot [45]. Untransformed and transformed coffee beans (T0) were powdered by grinding the coffee seeds with the use of liquid N_2. _Proteins from the seed powder were extracted at 4°C with four volumes of 150 mM succinic acid buffer pH 5.0, containing 60 mM NaCl, 20 mM CaCl_2 _and 10 mM sodium metabisulfite. The extract was slowly agitated for 60 min at 4°C and centrifuged at 10,000 × *g *for 20 min, and the supernatant was transferred to new Eppendorf tube. Protein extracts were quantified by the Bradford protein assay (Bio-Rad) according to the manufacturer's instructions. Five micrograms of protein from each transformed plant was separated using 15% SDS-PAGE (20 mA, 90 min). About 400 ng of semi-purified α-AI1 from *P. vulgaris *was used as a positive control, and 5 μg of seed protein extract from the untransformed coffee plant used as a negative control. Another SDS-PAGE was run with 10 μg of protein extract and stained with Coomassie Blue [46]. Proteins of the first SDS-PAGE were transferred to a nitrocellulose membrane (Hybond-C Extra, Amersham Biosciences) using Trans-Blot^® ^SD Semi-Dry (Bio-Rad), following the manufacturer's instructions. After transfer to the nitrocellulose membrane, the membrane was blocked with a TBS buffer containing 3% BSA and then incubated with an anti-α-AI1 polyclonal antibody produced in rabbit. Final detection of the α-AI1 protein was made with an Anti-Rabbit IgG conjugate to alkaline phosphatase (SIGMA), and the reactive proteins in the nitrocellulose membrane were revealed with a Kit AP Conjugate Substrate (Bio-Rad), according to the manufacturer's instructions.

### Quantification of expressed α-AI1 using ELISA

α-AI1 was quantified by ELISA assays [47] with 2.5 μg of the seed protein from each one of the 3 transformed coffee plants (T0) and one from the non-transgenic coffee plant. Assays were performed in triplicate on a 96-well Eia/Ria microplate (Sigma-Aldrich) with the same antibodies previously used in the Western blot analysis.

### *Hyphotenemus hampei α*-amylase inhibition assay

The inhibition of *H. hampei α*-amylases by the α-AI1 expressed in transgenic *C. arabica *plants (T0) was measured using the dinitrosalicylic acid (DNS) method, adapted from Bernfeld [48], using 1% soluble starch as a substrate. A protein extract from *H. hampei *adult insects was prepared with 150 mM succinic acid buffer pH 5.0, containing 60 mM NaCl, 20 mM CaCl_2 _and 10 mM sodium metabisulfite. Coffee seed protein extracts were made using the same buffer solution. The coffee protein extract was dialysed against the extraction buffer using a Mini Dialysis Kit (1 kDa cut-off) (Amersham Biosciences) to eliminate soluble sugars. The reaction was run using one unit of *H. hampei α*-amylase and 30 μg of coffee seed protein extracts. Assays were carried out in triplicate.

## Authors' contributions

AEADB performed the characterisation of transformed plants and wrote the manuscript. ÉVSA carried out the plant transformation experiment. MCS contributed to the ELISA experiments. AV, DLS, OBON and TLR revised the manuscript. MFGS conceived and coordinated the study, performed experiments, and helped write and correct the manuscript. All authors have read and approved the final manuscript.
